# Differential Lipid Dependence of the Function of Bacterial Sodium Channels

**DOI:** 10.1371/journal.pone.0061216

**Published:** 2013-04-08

**Authors:** Nazzareno D'Avanzo, Emily C. McCusker, Andrew M. Powl, Andrew J. Miles, Colin G. Nichols, B. A. Wallace

**Affiliations:** 1 Department of Physiology and GEPROM (Group d'étude des Proteins Membranaires), Université de Montréal, Montréal, Québec, Canada; 2 Department of Biological Sciences, Institute of Structural and Molecular Biology, Birkbeck College, University of London, London, United Kingdom; 3 Department of Cell Biology and Physiology and Center for Investigation of Membrane Excitability Diseases, Washington University School of Medicine, St. Louis, Missouri, United States of America; University of Montreal, United Kingdom

## Abstract

The lipid bilayer is important for maintaining the integrity of cellular compartments and plays a vital role in providing the hydrophobic and charged interactions necessary for membrane protein structure, conformational flexibility and function. To directly assess the lipid dependence of activity for voltage-gated sodium channels, we compared the activity of three bacterial sodium channel homologues (NaChBac, NavMs, and NavSp) by cumulative ^22^Na^+^ uptake into proteoliposomes containing a 3∶1 ratio of 1-palmitoyl 2-oleoyl phosphatidylethanolamine and different “guest” glycerophospholipids. We observed a unique lipid profile for each channel tested. NavMs and NavSp showed strong preference for different negatively-charged lipids (phosphatidylinositol and phosphatidylglycerol, respectively), whilst NaChBac exhibited a more modest variation with lipid type. To investigate the molecular bases of these differences we used synchrotron radiation circular dichroism spectroscopy to compare structures in liposomes of different composition, and molecular modeling and electrostatics calculations to rationalize the functional differences seen. We then examined pore-only constructs (with voltage sensor subdomains removed) and found that in these channels the lipid specificity was drastically reduced, suggesting that the specific lipid influences on voltage-gated sodium channels arise primarily from their abilities to interact with the voltage-sensing subdomains.

## Introduction

Voltage-gated sodium channels (VGSC) are membrane proteins found in a wide range of human tissues, including muscle and central and peripheral nerves. Cells in these tissues exhibit considerable variation in the lipid contents of their plasma membranes. VGSCs are responsible for the rapid electrical signaling important for the initiation of the action potential in excitable cells and for maintaining homeostasis in a variety of eukaryotic organisms [1], [2]; they are pharmaceutical targets for the treatment of a wide variety of diseases. VGSCs have also been identified in a number of prokaryotic organisms where they may have roles in motility and chemotaxis functions [3], [4].

All VGSCs exhibit a common pore-forming architecture composed of two spatially-separated but structurally linked two transmembrane helix pore domains and four transmembrane helix voltage-sensor domains (VSDs). Eukaryotic VGSCs are single polypeptide proteins with molecular weights of >200 kDa, each comprised of four pseudo-repeats of the VSD-pore motifs [5]. In contrast, bacterial VGSCs are comprised of single VSD-pore subunits, four of which associate to form a tetramer of approximately the same size as the eukaryotic VGSC [6]. Recombinant expression of a number of bacterial VGSC homologues in mammalian cells shows that, like eukaryotic sodium channels, they are highly selective for Na^+^, bind channel-blocking drugs and exhibit voltage-dependent activation, inactivation and recovery [7]–[9]. More than 30 prokaryotic sodium channel homologues have thus far been identified from bacteria that occupy a wide range of ecological niches. The channels exhibit high levels (16–65%) of sequence identity, but have important differences that may contribute to different functional characteristics and lipid sensitivities.

Lipid bilayers are important for maintaining the integrity of cellular compartments but also play vital roles in stabilizing membrane proteins at specific cellular locations. Lipids can influence membrane protein function either directly by specifically associating with the protein or indirectly by altering the fluidity or electrochemical environment of the protein. Their hydrophobic fatty acid chains and charged head groups provide interactions that can be important for channel structure, flexibility and function. Accordingly, it is well established that many membrane proteins require specific lipids to function [10]–[16] and that charged phospholipids are essential for properly functioning of ion channels [13], [17], [18]. Electrophysiological studies have shown that eukaryotic sodium channel functions vary with membrane lipid composition: Hypercholesterolemia results in decreased current density and increased inactivation of cardiac VGSCs [19]. Nav1.4 channels appear to be regulated by cholesterol through changes in the elastic properties of the bilayer [20], while β-adrenergic stimulation of Nav1.5 requires partitioning into cholesterol-rich lipid rafts [21], [22].

Biochemical and structural data indicate that voltage-sensing of voltage-gated cation channels depends on spatially-separate VSDs, joined only to the pore domains by single polypeptide linkers, and that they are highly exposed to the lipid bilayer. Indeed, lipids have even been observed between adjacent VSDs and between the pore and VSD in the crystal structures of the Kv1.2/2.1 potassium channel [23] and the NavAb [24], [25] and NavRh sodium channels [26]. Despite the intimate relationship that exists between VGSCs and the membranes in which they are embedded, little information currently exists regarding the specificity and molecular interactions that affect their structure and function.

In this study, we examined several bacterial VSGC homologues from different species reconstituted into model liposomes of defined compositions in order to gain insight into how membrane lipids may contribute to the function and structure of these channels.

## Results and Discussion

### Purification and Isolation of Tetrameric NaChBac, NavMs and NavSp

**Figure 1:**
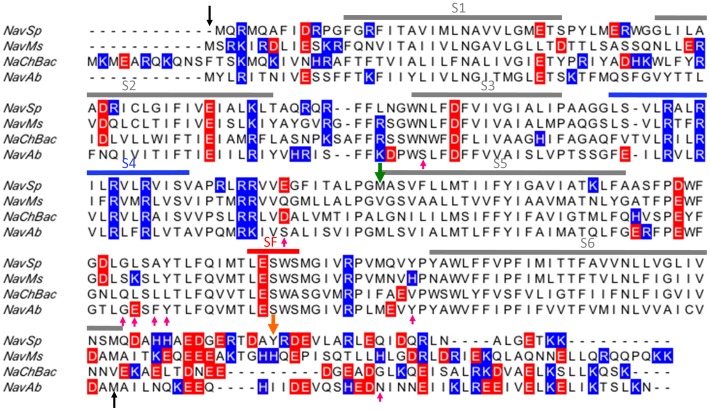
Sequence alignment of bacterial voltage-gated sodium channels. The black arrows indicate the region visible in the NavAb crystal structure (PDBID 3RVY) which was used to create homology models of each channel. Positively charged residues are shown in blue, negatively charged residues in red. The pink upward arrows indicate residues within 5 Å of the phosphocholine headgroups observed in the NavAb crystal structures (PDBID 4EKW or 3RVY). The green downward arrow indicates the start of the pore-only constructs and the orange downward arrow indicates the site of the C-terminal truncation of the NavMs constructs.

**Figure 2:**
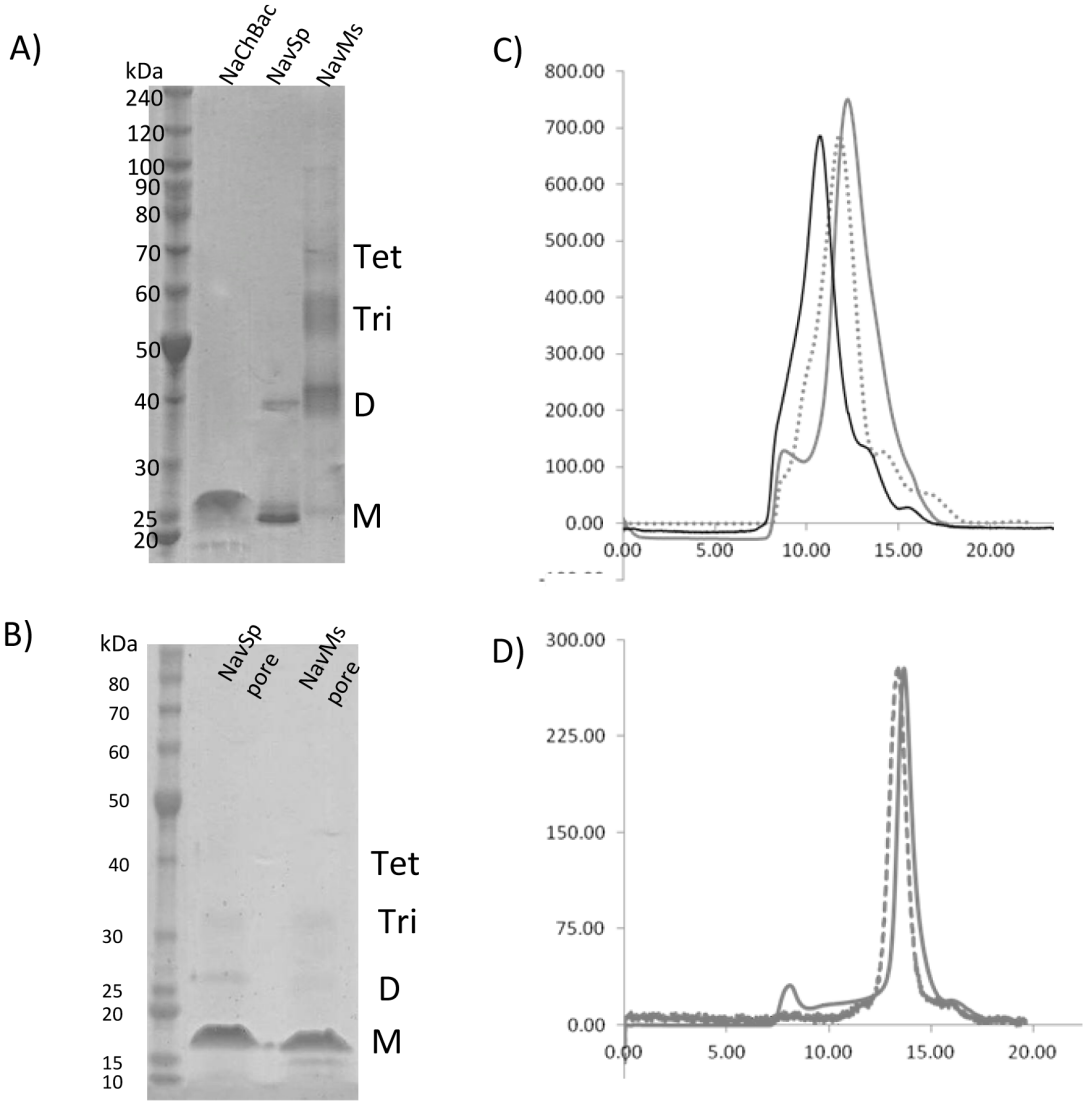
Purification of NaChBac, NavSp and NavMs channels. (**A**) SDS-PAGE of purified proteins in Cymal-5. The positions of the predicted monomeric (M), dimeric (D), trimeric (Tri) and tetrameric (Tet) forms are indicated. (**B**) SDS-PAGE of purified pore-only proteins in DM. The position of the predicted monomeric (M), dimeric (D), trimeric (Tri) and tetrameric (Tet) forms are indicated. (**C**) Gel filtration chromatography profiles for NaChBac (black solid), NavSp (grey dashed) and NavMs (grey solid). The predominant peak corresponds to the molecular mass of tetrameric voltage-gated sodium channel plus Cymal-5 micelle. (**D**) Gel filtration chromatography profiles for NavSp pore (grey dashed) and NavMs pore (grey solid). The predominant peak corresponds to the molecular mass of tetrameric voltage-gated sodium channel plus DM micelle.

Full-length sodium channels from *B. haldurans* (NaChBac) and *S. pomeroyi* (NavSp) and a partially C-terminal truncated channel from *M. spirillium* (NavMs) (Figure 1) were purified in Cymal-5 and the pore-only constructs of NavMs and NavSp were purified in decyl maltoside (DM). The full-length NaChBac and NavSp channels migrated in SDS denaturing gels at positions corresponding to slightly smaller than their predicted monomeric molecular masses (NaChBac ∼31 kDa, NavSp ∼29 kDa) (Figure 2A), as is commonly observed for the prokaryotic VGSCs [27], [28]. Similarly, NavMs and NavSp pores migrated primarily at their predicted monomeric molecular mass (pre-histidine tag cleavage NavSp pore ∼16.9 kDa and NavMs pore ∼14.3 kDa) (Figure 2B). The NavMs channel migrated predominantly at its predicted dimeric molecular mass (Figure 2A) but also produced bands which Western Blot analyses (data not shown) indicated were monomers (∼26.5 kDa), trimers and tetramers. Size exclusion chromatography was used to identify the native oligomeric species present in solution. All channel constructs eluted in (native) gel filtration chromatography predominantly at the predicted tetrameric molecular mass plus the approximate mass of the micelle (Cymal-5 ∼23 kDa or DM ∼30 kDa): [NaChBac ∼126 kDa, NavSp ∼116 kDa, NavMs ∼106 kDa (Figure 2C)], as did the NavSp pore [∼59 kDa and NavMs pore ∼50 kDa (Figure 2D)].

### Lipid Dependence of Sodium Flux in Bacterial Sodium Channels

**Figure 3:**
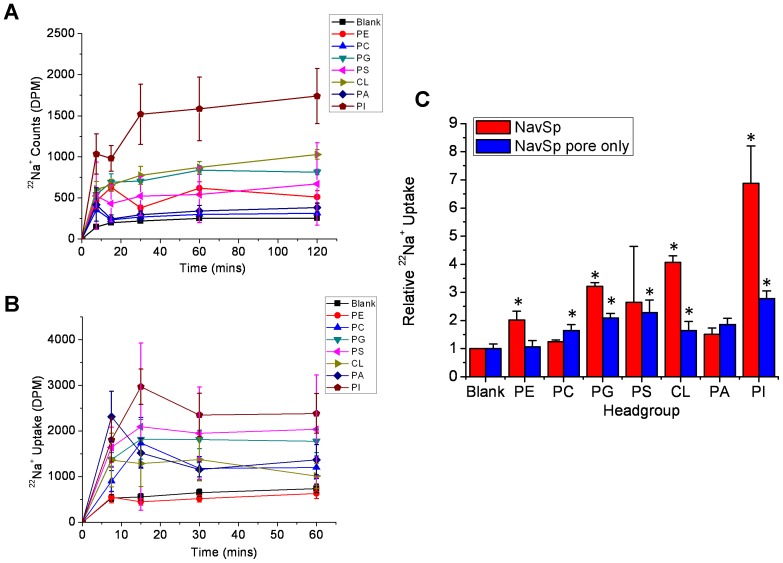
Lipid dependence of purified NavSp channels. ^22^Na^+^ uptake counts of full-length (**A**) or pore-only (**B**) constructs of NavSp channels in liposomes composed of POPE and the indicated lipid. (**C**) Relative ^22^Na^+^ uptake of NavSp at 120 mins normalized to the uptake into liposomes that do not contain NavSp protein (ie. “Blank”) (n = 3 and 6, respectively, +/− s.e.m.).

**Figure 4:**
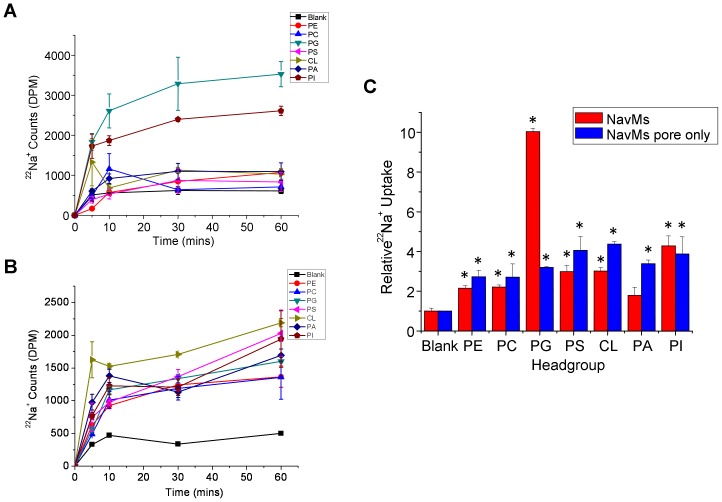
Lipid dependence of purified NavMs channels. ^22^Na^+^ uptake counts of full-length (**A**) or pore-only (**B**) constructs of NavMs channels in liposomes composed of POPE and the indicated lipid. (**C**) Relative ^22^Na^+^ uptake of NavMs at 60 mins normalized to uptake into liposomes that do not contain NavMs protein (n = 4 and 9, respectively, +/− s.e.m.).

**Figure 5:**
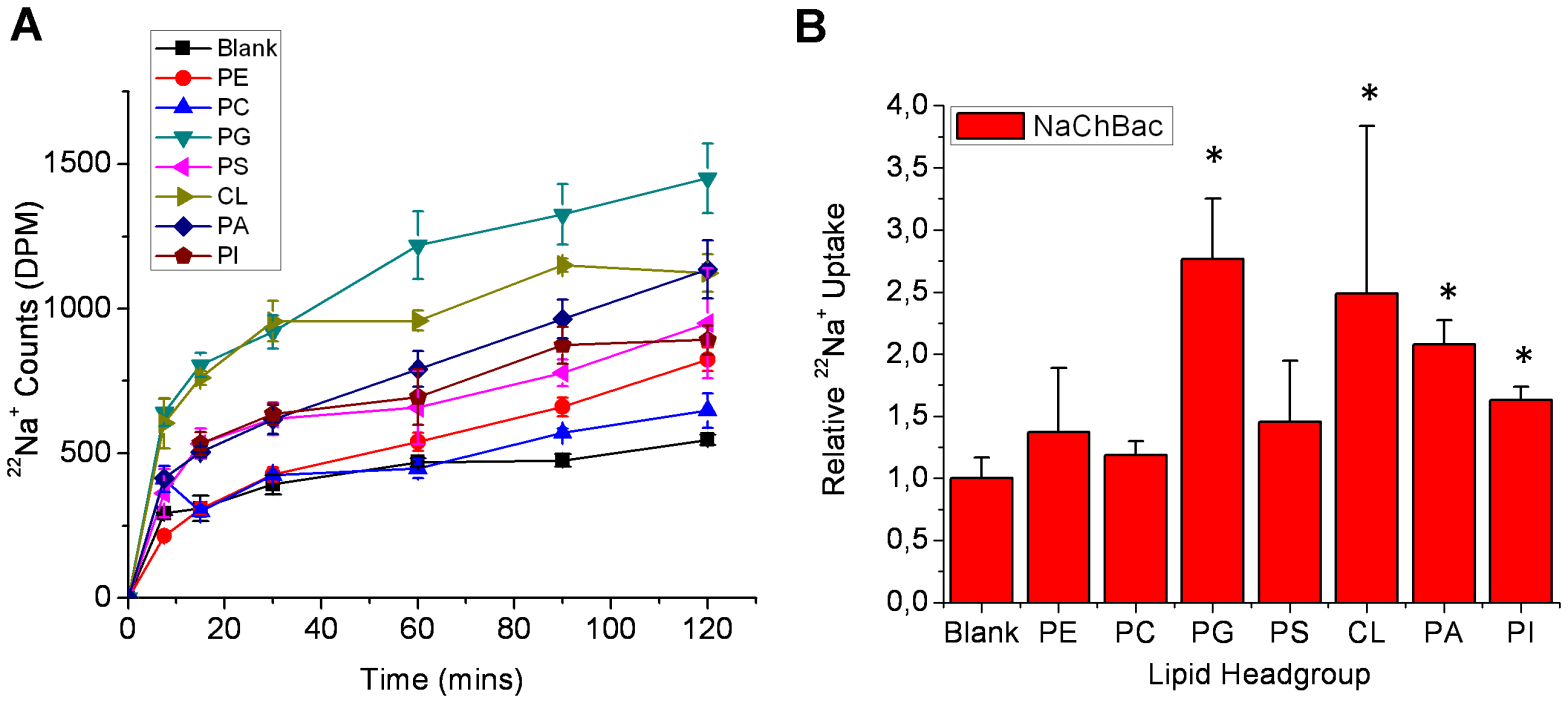
Lipid dependence of purified NaChBac channels. (**A**) ^22^Na^+^ uptake counts of NaChBac channels in liposomes composed of POPE and the indicated lipid. (**B**) Relative ^22^Na^+^ uptake of NaChBac at 120 mins normalized to uptake into liposomes that do not contain NaChBac protein (n = 6, +/− s.e.m.).

The same three bacterial sodium channels (NavSp, NavMs, NaChBac) were reconstituted into liposomes containing a 3∶1 ratio of the zwitterionic lipid 1-palmitoyl 2-oleoyl phosphatidylethanolamine (POPE), and a “guest” glycerophospholipid with head groups that were either charged [phosphatidic acid (PA), phosphatidyl glycerol (PG), phosphatidyl serine (PS), cardiolipin (CL) and phosphatidylinositol (PI)] or zwitterionic [phosphatidyl choline (PC)]. Channel function was assessed by measuring cumulative ^22^Na^+^ into the proteoliposomes (Figures 3, 4, 5). A unique lipid dependence profile was obtained for each homologue. Common to all three channels was low specific activity (above background uptake into protein-free liposomes) in liposomes composed only of zwitterionic lipids such as PE and PC (Figures 3, 4, 5). NavSp exhibited the greatest activity when negatively-charged PI lipid was included (Figure 3). NavMs and NaChBac also showed maximal activity with a negatively-charged lipid, but in this case, PG (a lipid with a smaller head group) was preferred (Figures 4 & 5). Notably, no activity above background was observed for NavMs or NavSp channels in liposomes containing PA only, while some activity was still observed for NaChBac under these conditions. Conversely, in liposomes containing PS, activity was observed for NavMs channels but there was no activity above background for NavSp and NaChBac channels.

Lipid-activation of channels could arise from a favorable shift in the equilibrium between the closed or inactivated states and the open state, which could be initiated by interactions with either the pore domain or the VSD. In both sodium and potassium channels the VSD and pore domain are loosely associated, tethered only by a short polypeptide; however, that linkage is crucial to the activation process [23]–[26], [29]. Bacterial sodium channels (in particular NavMs and NavSp) have been shown to be functional, even in the absence of their VSDs, with pore-only constructs showing activity when reconstituted into liposomes or bilayers [27], [30], [31]. To assess whether the phospholipid dependence of bacterial sodium channels depends on lipid interactions with the pore or the VSD, ^22^Na^+^ uptake experiments using pore-only constructs of NavSp and NavMs, the two homologues which had demonstrated the highest sensitivities to the presence of negatively charged lipids, were performed. The pore-only NavMs construct was insensitive to lipid composition, with the exception of a small but significant increase in activity when CL was present in the liposomes (Figure 4). The pore-only NavSp construct did have some lipid dependence, with greatest activity in the presence of PG, PS and PI, but this effect was drastically lower than that observed for full-length NavSp protein (Figure 3). These data suggest that the lipid dependences of sodium channels may arise primarily from their abilities to effect changes in the VSDs, which are then transmitted to modify the open/closed/inactivated equilibria of the pore domains, and seem to arise from specific lipid head group interactions that are uniquely favorable to the different channel homologues.

### PI(4,5)P_2_ and Bacterial Voltage-Gated Sodium Channels

**Figure 6:**
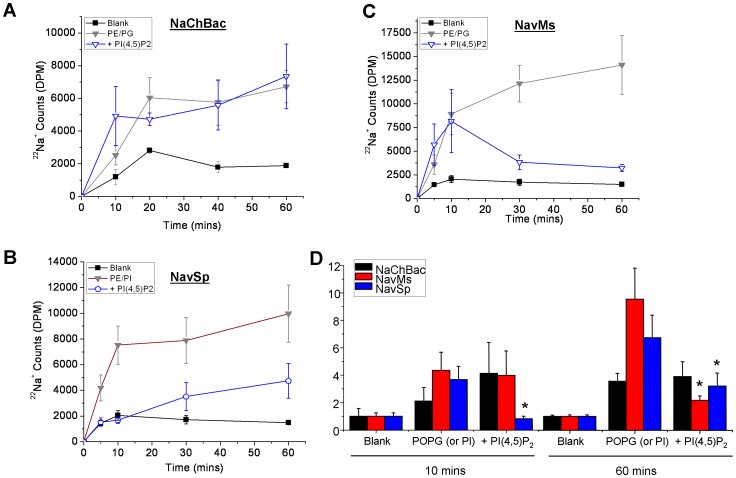
Effect of PI(4,5)P_2_ on bacterial voltage-gated sodium channels. (**A–C**) Time-dependent uptake of ^22^Na^+^ into proteoliposomes containing 3∶1 POPE∶POPG (or PI in the case of NavSp) plus 1% PI(4,5)P_2_ and full-length NaChBac, NavMs, or NavSp proteins, respectively. (**D**) Relative ^22^Na^+^ uptake at 10 mins and 60 mins from the time course measurements in parts A–C indicate that NaChBac channels are unaffected by the presence of 1% PI(4,5)P_2_, NavSp channels are approximately 50% inhibited by 1% PI(4,5)P_2_ and NavMs channels have complex regulation by PI(4,5)P_2_ (* P<0.05).

Arguably the best characterized lipid modulator of ion channel activity, is phosphatidylinositol 4,5-bisphosphate (PI(4,5)P_2_) [32]. To determine if PI(4,5)P_2_ acts as a modulator of bacterial VGSCs, channel proteins were reconstituted into liposomes containing 3∶1 PE/PG (for NaChBac and NavMs) or PE/PI (for NavSp) with or without 1% PI(4,5)P_2_ and ^22^Na^+^ uptake was assessed (Figure 6A–C). While NaChBac was insensitive to PI(4,5)P_2_ (Figure 6D), NavSp channels showed a marked inhibition in the presence of 1% PI(4,5)P_2_ (Figure 6 D). PI(4,5)P_2_ inhibition has not been reported for eukaryotic Na channels, and it is intriguing that while eukaryotic Kir channels are actually activated by PI(4,5)P_2_, bacterial Kir channels also show a unique inhibition by PI(4,5)P_2_ [14], [33], [34] which may reflect the lack of PI(4,5)P_2_ in bacterial membranes and ubiquitous presence in eukaryotic membranes [12], [14]. On the other hand, the time-course of NavMs protein in the presence of PI(4,5)P_2_ shows a peak followed by a decline (Figure 6C), and may suggest activation by this lipid. A complex phenomenon has also been observed for ^86^Rb^+^ uptake through K^+^ channels when channel activity is exceedingly high [35].

### Synchrotron Radiation Circular Dichroism (SRCD) Spectroscopy of Channels in Liposomes

To investigate whether differences in channel function in different lipids were reflected in overall changes in the structure of the protein, SRCD spectroscopy was used to examine full-length NavSp, the protein that was most sensitive to lipid effects. The spectral properties of NavSp were examined in liposomes composed of PE and the three guest lipids (PC, PG, and PI) that produced the largest differences in flux behavior. SRCD was used instead of conventional CD spectroscopy for several reasons [36]: 1) the high flux of the synchrotron light source results in signal-to-noise ratios that are much greater than in a lab based instrument, meaning that more subtle differences can be detected; 2) the lower wavelengths measureable (in this case down to 180 nm) permit investigation of more spectral transitions and thus the data have a higher information content that would enable detection of more features that might change; and 3) the sample geometry of an SRCD beamline enables examination of scattering samples such as liposomes without spectral distortion.

**Figure 7:**
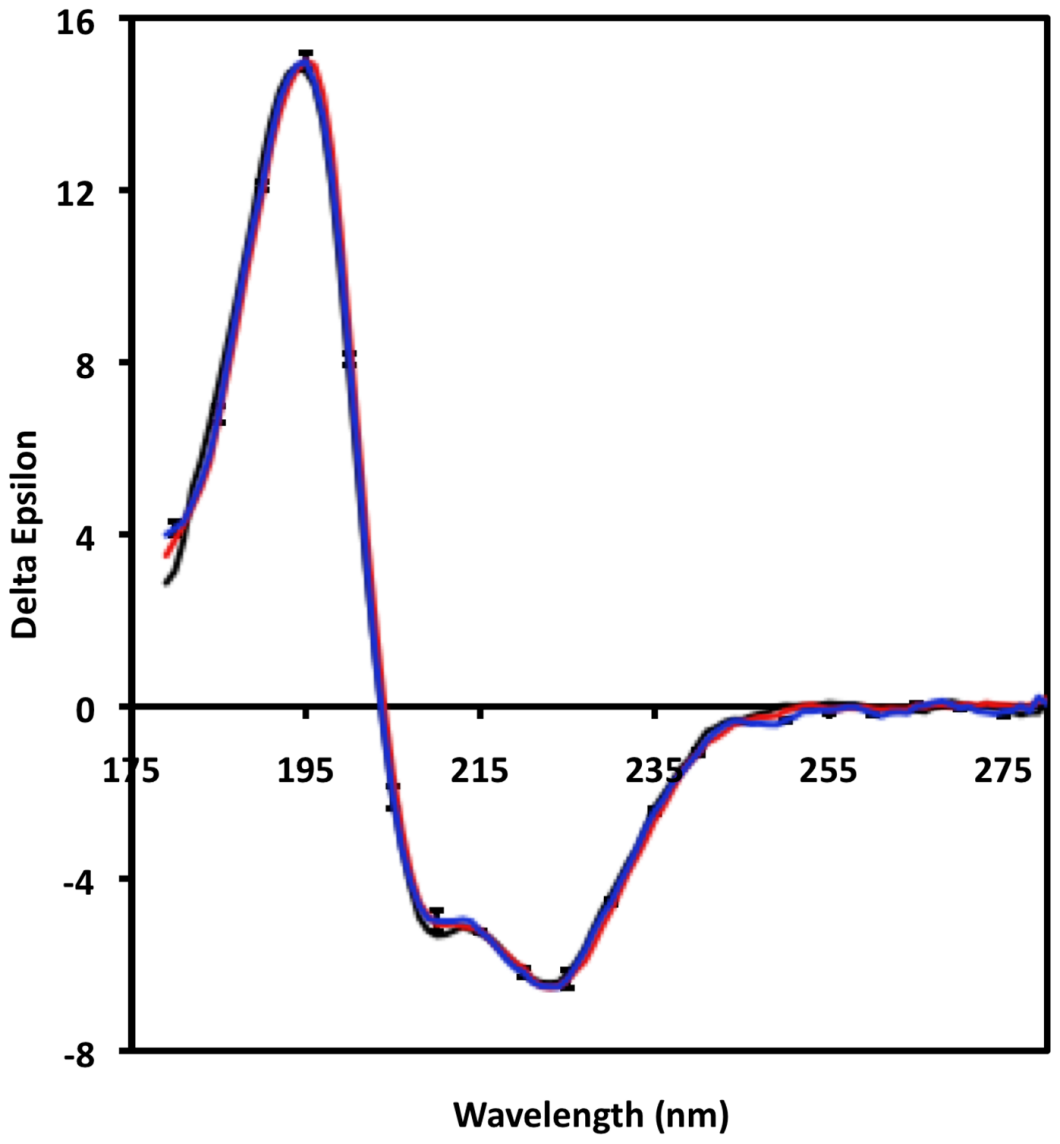
SRCD spectra of NavSp in liposomes of different lipid compositions. The “guest” lipids were PG (black), PC (red), and PI (blue). The error bars represent one standard deviation in the replicate measurements of spectra.

There are no obvious differences between the NavSp spectra in the three types of liposomes relative to the variations between repeat measurements for the NavSp homologue in the three types of samples (Figure 7). The error bars range from <1% of the signal at 190 nm to 2% of the signal at 220 nm, suggesting that any differences in net structure must be minimal. Indeed, the data can then be used to calculate the net secondary structure of each sample (Table 1); in the three samples, the secondary structures (eg. helix content) differ by ≤2%, well within the standard deviations of the calculated values (1–3%). For a protein comprised of 258 amino acids, even assuming a 2% difference was real, this would correspond to a net difference in structure for 5 amino acids at most and since the differences are within the significance levels of the measurements, there is high confidence that the observed variations in functional properties are not reflected in local refolding of the channel secondary structure. Significant changes could occur in its tertiary structure, as was seen for the open and closed conformations of the voltage-gated sodium channel [30], with no changes in secondary structure content, but the low wavelength SRCD data which are sensitive to such changes, do not suggest any such refolding of the structure.

**Table 1:** Calculated secondary structure of the NavSp channel in liposomes of different compositions based on SRCD measurements.

	“Guest “ Lipids		
	PC	PG	PI
**Helix**	68±2	66±1	67±3
**Sheet**	4±1	7±2	6±2
**Unordered**	17±4	17±3	17±3

The percentage values listed are the average secondary structures calculated from several methods of analyses applied to the same data sets (the +/− values indicate the variations between algorithms). All liposomes include PE; the “guest” lipid is the second lipid present in the liposome sample.

**Figure 8:**
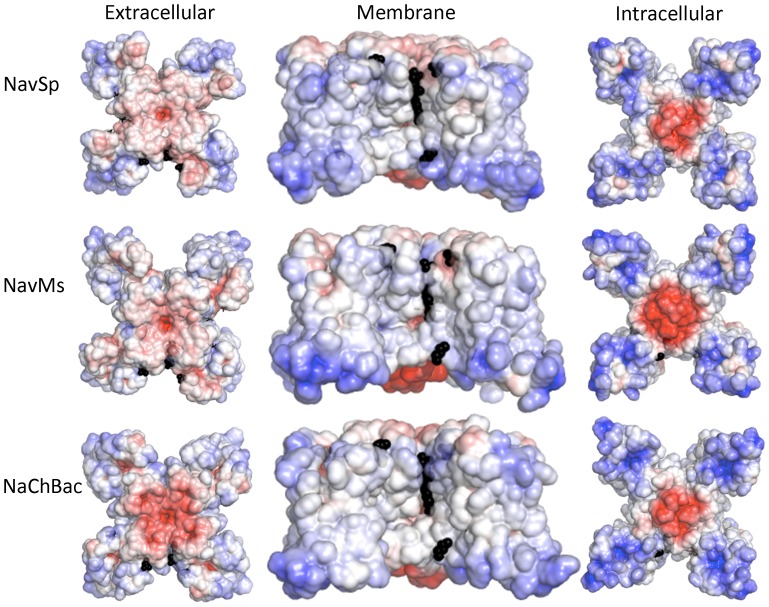
Model structures of the three homologues. Comparison of the electrostatic potentials at +/−5 KT/e plotted on the solvent-accessible surfaces of (**A**) NavSp, (**B**) NavMs and (**C**) NaChBac, based on homology models constructed from NavAb in the activated closed state (PDBID:3RVY), viewed along the extracellular surface (left panel), the membrane normal (middle panel) and from the cytoplasmic surface (right panel). The black spheres indicate the positions of ordered lipids in the original NavAb (PDBID: 3RVY) crystal structure used to produce the models.

**Figure 9:**
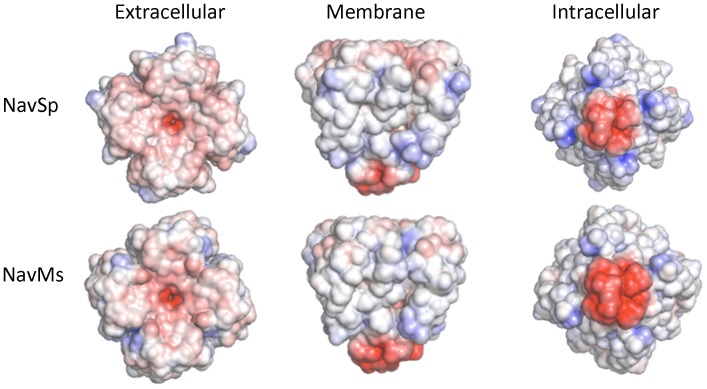
Model structures of the pore-only constructs of NavSp and NavMs. Comparison of the electrostatic surface potentials (as in Figure 6) of the pore-only constructs of NavMs and NavSp. The NavMs is the crystal structure of the NavMs pore (PDBID: 4F4L) and the NavSp is a homology model based on that crystal structure.

These SRCD studies effectively eliminate the possibility of significant refolding of the polypeptide chains in different lipids as being the source of the different sensitivities and limit the magnitude and types of structural changes, if any, that occur, suggesting that the more likely sources of the differences are specific interactions between the lipid molecules resulting from their steric or electrostatic properties. Indeed, examination of the electrostatic surfaces of the different homologues (Figure 8) as well as of the pore-only constructs (Figure 9), suggest these may be an important determinant in the lipid interactions.

### Modelling and Electrostatic Calculations on Channel and Pore Homologues

The aligned sequences (Figure 1) of the different homologues provide no obvious structural basis for differential lipid sensitivities, such as specific individual charges in particular locations of the extramembranous regions of the protein. We therefore generated three-dimensional models of each homologue and their electrostatic surfaces were compared (Figure 8). In each channel, the peripheries of the extracellular surfaces were mostly uncharged, but the peripheries of the intracellular surfaces exhibit strongly positive electrostatic potentials, with the charge distributions of NavSp and NavMs differing somewhat from that of NaChBac in the regions of the VSDs. These electrostatic differences might account for the lipid sensitivities. It is interesting that some of the residues within 5 Å of the lipid choline headgroups observed in the NavAb crystal structure [25] are not highly conserved in the sequences of NaChBac, NavSp and NavMs channels (Figure 1), although there is no obvious pattern of changes to charged residues at these sites. It is tempting to speculate that such differences in sequence at these regions (and elsewhere) in the channel may contribute to different sensitivities to membrane lipids among various VGSCs homologues. Most interestingly, the electrostatic surfaces on the peripheries of the pore-only constructs are nearly all neutral (Figure 9), consistent with removal of the charged regions of the VSD eliminating the sites for specific charged lipid interactions, and hence suggesting an activation mechanism that includes lipid-VSD interactions that are transmitted to the pore domains in a way that modulates their activation mechanism.

### Relation to Physiological Regulation by Membrane Lipids

In this study we have examined the lipid dependence of the bacterial VGSC homologues NavSp, NavMs, and NaChBac, using purified proteins reconstituted into liposomes of defined composition. For each channel, we observed low levels of activity above background in the zwitterionic lipids PE and PC, but differing sensitivities to negatively-charged lipids. Both gram-positive and gram-negative bacterial membranes have been shown to primarily contain PE, PG, and CL lipids in both leaflets - although asymmetrically - with PS, PA and PC lipids found to also occur in some bacteria [37]–[41]. PI lipids are essential to eukaryotic cells, but rarely found in prokaryotes, generally confined to actinomycetes, myxobacteria, and a select few other bacteria [39]. Hence, although we cannot directly correlate the observed sensitivities with specific lipid contents of the host bacteria, it is likely that the mixture of lipids present in each host can contribute to the functional properties of the particular homologue present.

NaChBac channels appeared insensitive to PI(4,5)P_2_ while NavSp channels are approximately 50% inhibited in the presence of 1% PI(4,5)P_2_ (Figure 6). PI(4,5)P_2_ has also not been identified in bacterial membranes. Thus, other than the sensitivities to PI and PI(4,5)P_2_, the lipid dependence we observe may be critical for the physiological function of these channels in their host bacterial strain. On the other hand, inward rectifier K^+^ channels KirBac1.1 from *Burkholderia pseudomallei* and KirBac3.1 from *Magnetospirillum magnetotacticum* are also sensitive to lipids not found in bacterial membranes (notably cholesterol, PI(4,5)P_2_ and other phosphoinositides), but provide mechanistic insights to the sensitivities of these lipids in their eukaryotic counterparts [14], [33], [34], [47]–[49]. Thus, despite not being directly physiologically relevant, the observed regulation of bacterial VGSCs by PI and PI(4,5)P_2_ may enable important biophysical insights to be derived in the future.

Eukaryotic VGSCs are critical for the function of a wide range of human tissues, including muscle and central and peripheral nerves, with some isoforms specifically expressed in particular tissue types. A brief examination of the lipid composition of several of these tissues makes it quite reasonable to speculate that lipid regulation of eukaryotic VGSCs may also play a critical role in channel function, with varying specificities to lipids for each isoform. For example, while central neurons (gray and white matter, and spinal cord) in which Nav1.2 is expressed contain approximate 25% PC, 35–40% PE, 14–23% PS, 2% PI and only trace amounts of CL [42], [43], the sarcolemma of a cardiomyocyte where Nav1.5 is the primary sodium channel contains 35–52% PC, 25–36% PE, 3–5% PS, 6% PI and 1–13% CL [44]–[46]. Furthermore, these lipid profiles can change with the development of disease. Thus, as we observed for prokaryotic VGSCs, it is likely that membrane lipids also play a critical role in regulating the function of eukaryotic VGSCs.

## Materials and Methods

### Materials

The VGSC genes isolated from *Bacillus halodurans*, *Silicibacter pomeroyi* and *Magnetococcus spirillium (strain MC-1)* were supplied by Prof. David E. Clapham (Howard Hughes Medical Institute, Children's Hospital, Harvard Medical School, Boston, MA) [7], [8]. Quick ligase, restriction enzymes and DH5α chemically competent cells were purchased from New England Biolabs. Syntheses of PCR primers and all DNA sequencing were performed by Eurofins MWG Operon. Ni-NTA and all DNA purification supplies were purchased from Qiagen, Ltd (UK). Thrombin and the pET15b vector were purchased from Novagen, Inc (EMD Chemicals, Darmstadt, Germany). Dodecyl maltoside (DDM), cymal-5 and decyl maltoside (DM) were obtained from Anatrace, Inc (OH, USA). Synthetic lipids POPE (1-palmitoyl 2-oleoyl phosphatidyl ethanolamine), POPG (1-palmitoyl 2-oleoyl phospatidyl glycerol), POPS (1-palmitoyl 2-oleoyl phosphatidyl serine), POPA (1-palmitoyl 2-oleoyl phosphatidic acid), POPC (1-palmitoyl 2-oleoyl phosphatidyl choline), EPC (1-palmitoyl 2-oleoyl-ethyl phosphatidyl choline), CL (18∶1 cardiolipin), natural PI (phosphatidyl inositol from bovine liver), PI(4)P (phosphatidylinositol 4-phosphate from porcine brain), and PI(4,5)P_2_ (phosphatidylinositol 4,5-bisphosphate from porcine brain) were purchased from Avanti Polar Lipids (AL,USA). Mibefradil was obtained from Sigma-Aldrich.

### Expression and Purification

The full-length voltage-gated sodium channels from *B. halodurans* (NaChBac), *S. pomeroyi* (NavSp), and *Magnetococcus spirillium (strain MC-1)* (NavMs) (the latter had a partially truncated C-terminus, the site of which is indicated by red arrow in Figure 1) were expressed in *E. coli* and purified as previously described [27], [50]. Briefly, membranes were solubilized in 1% DDM and Ni-NTA purified in 0.25% Cymal-5. Thrombin was used to cleave the hexa-histidine tag at room temperature overnight at 5 units/mg protein. The cleaved his-tag and remaining his-tagged protein were incubated with Ni-NTA and the flow-through was collected. The Ni-NTA flow-through was filtered (0.22 µm filter) and concentrated before loading onto a gel filtration column (Superdex 200 10/300 GL, GE Healthcare). Protein was eluted with 10 mM Tris-HCl, pH 7.5, 100 mM NaCl, 0.02% NaN_3_ and 0.25% Cymal-5. The molecular biology, expression and purification of the pore-only constructs from *S. pomeroyi* and *Magnetococcus spirillium (strain MC-1)* were described previously [27], [30] (the green arrow in Figure 1 indicates the beginning of the constructs). The pore-only constructs were purified in 0.2% DM.

### ^22^Na^+^ Uptake Assay

Experiments were performed as previously described [27], [30]. 20–30 µg of each full-length or pore-only channel protein were added to 1 mg of phospholipid solubilized in buffer A (450 mM NaCl, 10 mM HEPES, 4 mM N-methyl-d-glucamine (NMG), 1 mM EDTA pH 7.5) and incubated for 30 min. Liposomes were formed by adding the protein/lipid sample to partially-dehydrated Sephadex G-50 columns presoaked in buffer A through polystyrene columns (Pierce Chemical Co.) and spinning to 2,500 rpm. The extraliposomal solution was exchanged by spinning the sample at 2,500 rpm through a second set of partially-dehydrated columns, now containing beads soaked in buffer B (400 mM sorbitol, 10 mM HEPES, 4 mM NMG, 1 mM EDTA pH 7.5). ^22^Na^+^ uptake was initiated by adding 400 µL of buffer B containing ^22^Na^+^ and when necessary, the indicated concentration of the channel blocker mibefradil. At various time points, 60 µL aliquots of the liposome uptake reaction were flowed through 0.5 ml Dowex cation exchange columns charged with NMGH^+^ to remove extraliposomal ^22^Na^+^. These aliquots were then mixed with scintillation fluid and counted in a liquid scintillation counter. Fractional flux at 60 minutes in the presence of mibefradil was determined following data subtraction of uptake counts measured at each time point in protein-free liposomes.

### Synchrotron Radiation Circular Dichroism Spectroscopy

Lipid mixtures (2.5 mg) containing 3∶1 POPE: “guest” lipid were dried down from chloroform solution onto the walls of a thin glass vial (where the guest lipid was either POPC, POPG or Liver PI). 1 ml of buffer (10 mM Tris-HCL, pH 7.8, 50 mM NaCl, 0.02% NaN_3_, 0.3% Cymal-5) was added. The sample was sonicated to optical clarity in a bath sonicator. NavSp protein (1 mg) was then added to each of the sonicated suspensions to give a 400∶1 molar ratio of lipid∶channel tetramer. Detergent was removed by the addition of 120 µl of Calbiosorb adsorbent beads (Calbiochem). The reconstituted vesicles were removed from the adsorbent beads and rapidly diluted into buffer (70 ml; 10 mM Tris-HCL, pH 7.8, 50 mM NaCl, 0.02% NaN_3_) to ensure complete removal of the detergent. Proteoliposomes were pelleted by centrifugation at 100,000× *g* at 18°C for 30 min and resuspended in 250 µl of the same buffer via gentle aspiration. The proteoliposomes were briefly sonicated in a bath sonicator and spun at 3,000× *g* for 1 min. A lipid blank was also prepared for each sample, following the same method without the addition of protein. For each sample, the concentration was determined from the A_280_ measured in triplicate on a Nanodrop 1000 UV spectrophotometer immediately before the SRCD spectrum was acquired.

SRCD data were collected on beamline CD1 at the ISA synchrotron located at the University of Aarhus, Denmark. Similar measurements were also undertaken at the CD12 beamline at the ANKA synchrotron in Karlsruhe, Germany and at the DISCO beamline at the Soleil Synchrotron in France. Protein samples at concentrations between 2 and 5 mg/ml were loaded into a 0.0015 cm pathlength quartz Suprasil cell (Hellma UK) and spectra measured at 25°C using a step size of 1 nm, and an averaging time of 2 s. Three replicate scans collected in the wavelength range from 280 nm to180 nm were averaged and the average of three replicate scans of the cognate baseline subtracted. Spectra were then calibrated to a spectrum of camphorsulfonic acid measured immediately after each beam fill, and scaled to units of delta epsilon using a mean residue weight of 114.4 All processing was carried out using CDTool software [51]. Secondary structure analyses were carried out using the DichroWeb analysis server [52]. Values from the algorithms CONTINLL [53], SELCON3 [54] and CDDSTR [54] (using reference dataset SMP180 (which includes membrane proteins [55]) were averaged.

Circular dichroism spectral and meta-data have been deposited in the Protein Circular Dichroism Data Bank under accession codes CD0004040000, CD0004041000, and CD0004042000, and will be released upon publication.

### Modelling of Homologue Structures

The transmembrane regions and adjoining loops of full length NaChBac, NavSp and NavMs were modelled using the crystal structure of voltage-gated sodium channel from *Arcobacter butzleri*, NavAb (PDBID 3RVY) captured in a hybrid activated-VSD/closed-pore conformation. Amino acid sequences corresponding to NaChBac (Uniprot ID Q9KCR8), NavSp (Uniprot ID F7IVA8) and NavMs (Uniprot ID AOL5S6) were aligned to NavAb (Uniprot ID A8EVM5) using ClustalW [56] and displayed using Jalview software [57]. SwissModeller was used to create homology models based on the alignment from the N-terminal region through the base of the S6-helix.The pore-only construct of NavSp was modeled using the crystal structure of the voltage-gated sodium channel pore from *Magnetococus spirillum*, NavMs (PDBID 4F4L) [27]. Electrostatic surfaces were calculated using APBS software [58] and displayed using Pymol [59].
